# The Neglect of Asepsis in Ward Work

**Published:** 1913-02-08

**Authors:** 


### PROBLEMS IN ADMINISTRATIVE MEDICINE.

**(Carefully thought-out Contributions to this Section will be welcome.)**

### The Neglect of Asepsis in Ward Work.

In a former article we dealt with the necessity for absolute cleanliness on the part of surgeons who undertake operations, and we referred to the modern view that, in the interests of institution, patient, and surgeon alike, it is advisable that the surgeon and his assistants should wear gloves. That view, however, is not merely the result of failures and misfortunes which have resulted from the disregard of perfect asepsis in the carrying out of the operations of major surgery. It is the outcome of the experience of years which has shown that by the neglect of precautions, however trivial they may seem, failure and fatality are hazarded. Perhaps the imperative necessity of conforming to the rule of scrupulous cleanliness, which is the essence of asepsis, is not so generally understood as it ought to be. At least it is a rule which one sees disregarded almost daily in our hospitals, not so much, or almost never, in the performance of major operations, but in the manipulative surgery that is done in the wards by dresser and nurse, and sometimes even by the surgeons or physicians themselves. A common complaint of surgeons used to be, and still is, that their colleagues on the medical side are too eager to "puncture"; the surgeon looks upon surgery as a means of cure, not merely as a method of diagnosis; and the best surgeon, admittedly, is he who has a clear conception of what he is likely to find when he cuts through the integuments, and not he who operates with a view to clearing up a diagnosis. This complaint is in a certain degree justified. How often do those who frequent the wards and watch what goes on in them observe a physician undertake a diagnostic operation without safeguarding himself and the patient by that strict prophylactic asepsis without which no operation is justifiable? We have ourselves witnessed the senior physician in a large hospital gratuitously undertake the performance of a slight surgical operation on one of his patients with such an absolute disregard of the essentials of asepsis that it is not to be wondered at that the slight wound suppurated. Similarly it is common enough to see in the medical wards minor operations, such as aspirations, blood examinations, scrapings, and injections, done without the safeguard of absolute cleanliness. It may be argued that the vast majority of these operative interferences are unattended by untoward results which may be directly ascribed to the want of asepsis. That is probably true, but it is just as well to remember that there are cases on record—and they are not so infrequent as many think—in which a trivial operation has been followed by misfortune owing to the overlooking of these essential safeguards.

Let us take the simplest, possibly, of all ward operations—a hypodermic injection. This is a little operation which is every day carried out by the nurse or sister: it is in its very essence a triviality. The patient feels a slight, barely distinguishable prick, a little lump is raised under his skin, and a few minutes later he gains the benefit of the injection. It is a method employed for numerous purposes: to stimulate, to soothe, to give rest and sleep, to maintain the strength of a flagging heart, to put in the circulation a drug which acts more quickly when injected than when administered by mouth. The technique of the operation is simple; to carry it out under strict aseptic precautions is perfectly easy, though it entails a little care and trouble. The solution to be injected is ready sterilised in sealed ampoules, or in tablet form in tubes; the needle and syringe are boiled in the steriliser and placed in a sterile dish containing distilled water or normal saline. The site of the injection is cleaned with a bit of lint dipped in ether, the nurse sterilises her hands, pinches up the patient's skin at the site of the injection with her thumb and index finger, takes the filled syringe with attached needle, and plunges the needle into the integument, and then slowly injects. When the needle is withdrawn she seals up the little puncture with a strip of sterilised plaster, cleans the needle and syringe, and puts them back in their place.

That is the technique, but how often is it followed in its entirety? The common practice is to neglect the preliminary boiling of needle and syringe, to omit entirely the washing of the hands, and to inject the patient with a syringe that has been used upon other patients in the ward, and that has been cleaned merely by drawing water through it after each injection. It is argued that no bad results follow in the majority of cases. But every hospital worker knows that bad results have followed. Killing recorded a series of eight consecutive cases of erysipelas occurring in the same ward in which the infection was directly traceable to one nurse who had not sterilised her hypodermic needle; another writer a series of five cases of localised infection due to a similar cause.

### How Administration may Assist Asepsis.

The possibility of infection is always present, and a disregard of the ordinary rules of asepsis is never to be condoned. The use of "personal" hypodermics should be sternly prohibited in the ward, and it should be insisted upon that half a dozen hypodermic outfits are kept in each ward, which shall only be used after proper precautions have been taken that there is no chance of contamination by uncleanliness. Every nurse and ward clerk must be taught that a preliminary washing of the hands is the indispensable adjunct to any operation, however trivial, that he or she may be called upon to undertake. Attention to this detail must result in improved conditions generally. It is now generally the rule that no dresser approaches his case for dressing in the wards without first of all scrupulously sterilising his hands; the percentage of septic cases in a hospital varies, as is well known, not merely with the care exercised by the surgeon, but—and this is in general a far more important point—with the different "firms" that he employs. A careless firm may be responsible for a 2-per-cent. rise in the number of septic cases in a ward; an ideally careful firm may reduce the percentage to the vanishing point if the surgeon is lucky enough to get clean cases to work upon.

Under modern conditions diagnostic operations—that is to say, operative procedures which have their object in elucidating some obscure condition—are perhaps much more frequent than they used to be, and they are carried out on both the medical and surgical sides. Let us take, for instance, the newer methods of diagnosis of brain conditions. One of the helpful aids to the physician is the analysis of fluid or material derived from direct puncture of the brain, the ventricles, or the spinal theca. "Brain puncture" is now extensively employed for diagnostic purposes abroad, so much indeed that some continental surgeons are beginning to protest against its indiscriminate employment. The technique here again is simple. A small portion of the patient's scalp is shaved and carefully cleaned; the operator, having carefully sterilised his hands and instruments, takes up an electric drill, the point of which he places vertically on the patient's scalp at the site of puncture. When the current is turned on this drill rapidly perforates scalp, underlying periosteum, and the two tables of the skull with the diploe between. When the operator feels that he has reached the inner side of the inner table the current is turned off and the drill removed. A blunt trocar is now placed in the scalp opening, the hole in the skull reached with its point, and the trocar thrust down through the dura into the brain. A suction syringe is inserted into the trocar, and the fluid or matter or brain substance withdrawn for examination. Finally, the trocar is withdrawn, and the tiny puncture hole sealed up under aseptic precautions. This is a comparatively simple operation, but the slightest disregard of the rules of asepsis may be—and let us add has been—followed by the most disastrous after-effects, such as purulent meningitis, thrombosis, and localised abscess. Only by never neglecting asepsis, and by additionally safeguarding patient and operator by the use of sterile gloves, can such mishaps be avoided. It is a calamity when a minor operative procedure entails the death of a patient, and one that should be carefully investigated by those competent to judge where the blame lies. For blame in such cases there must be. We all recognise that there is an ever present element of luck, but it is well also to realise that most failures now ascribed to it might have been obviated if care had been taken.

The policy of perfection may be sneered at, but it is the only kind of policy that it pays a hospital to possess. And the way to ensure its smooth and efficient working is to educate the nursing staff to the urgent necessity of attending to the trivialities of aseptic surgery. The dressers may be left to the respective heads of their firms; they will become doctors, and find out that it is in their interests in every way to take care that they are never guilty of neglect. But there are still breaches in the training of the nurse. Too much nowadays is left to the theatre or the ward sister, whereas it should be the rule that the probationer must have the knowledge and the skill to do the simple technique of preliminary sterilising without a hitch, and with the full knowledge of the why and the wherefore of each step. The best way to ensure this is to train the nursing staff, by lectures and demonstrations, in the same way as the medical student is trained, and to insist that no manipulative treatment, however trivial it may seem, and whether or not its carrying out means a breach of the patient's skin, shall be conducted without scrupulous attention.

